# Within-host temporal fluctuations of *Trypanosoma cruzi* discrete typing units: the case of the wild reservoir rodent *Octodon degus*

**DOI:** 10.1186/s13071-017-2314-2

**Published:** 2017-08-07

**Authors:** Gemma Rojo, Alejandra Sandoval-Rodríguez, Angélica López, Sylvia Ortiz, Juana P. Correa, Miguel Saavedra, Carezza Botto-Mahan, Pedro E. Cattan, Aldo Solari

**Affiliations:** 1 Programa de Biología Celular y Molecular, ICBM, Facultad de Medicina, Universidad de Chile, Santiago, Chile; 2 Laboratorio de Parasitología Básico-Clínica, ICBM, Facultad de Medicina, Universidad de Chile, Santiago, Chile; 3 Departamento de Ciencias Ecológicas, Facultad de Ciencias, Universidad de Chile, Santiago, Chile; 4 Laboratorio de Ecología, Departamento de Ciencias Biológicas Animales, Facultad de Ciencias Veterinarias y Pecuarias, Universidad de Chile, Santiago, Chile

**Keywords:** *Trypanosoma cruzi*, *Octodon degus*, Xenodiagnosis, Triatomine species, qPCR

## Abstract

**Background:**

Chagas disease caused by *Trypanosoma cruzi* is considered a major public health problem in America. After an acute phase the disease changes to a chronic phase with very low parasitemia. The parasite presents high genetic variability with seven discrete typing units (DTUs): TcI-TcVI and Tc bat. The aim of this work is to evaluate fluctuation of parasitemia and *T. cruzi* DTUs in naturally infected *Octodon degus.*

**Methods:**

After animal capture parasitemia was obtained by qPCR and later the animals were evaluated by three serial xenodiagnoses using two insect vector species, *Mepraia spinolai* and *Triatoma infestans*. The parasites amplified over time by insect xenodiagnosis were analyzed by conventional PCR and after that the infective *T. cruzi* were characterized by means of hybridization tests.

**Results:**

The determination of *O. degus* parasitemia before serial xenodiagnosis by qPCR reveals a great heterogeneity from 1 to 812 parasite equivalents/ml in the blood stream. The *T. cruzi* DTU composition in 23 analyzed animals by xenodiagnosis oscillated from mixed infections with different DTUs to infections without DTU identification or vice versa, this is equivalent to 50% of the studied animals. Detection of triatomine infection and composition of *T. cruzi* DTUs was achieved more efficiently 40 days post-infection rather than after 80 or 120 days.

**Conclusion:**

*Trypanosoma cruzi* DTUs composition fluctuates over time in naturally infected *O. degus.* Three replicates of serial xenodiagnosis confirmed that living parasites have been studied. Our results allow us to confirm that *M. spinolai* and *T. infestans* are equally competent to maintain *T. cruzi* DTUs since similar results of infection were obtained after xenodiagnosis procedure.

## Background

Chagas disease is a vector-borne disease caused by the flagellated parasite *Trypanosoma cruzi*, and transmitted via triatomine insects (Hemiptera: Reduviidae) to several mammal species [1]. The infection caused by *T. cruzi* presents two phases, an acute and a chronic one. For both domestic and wild transmission cycles the infection of mammal hosts occurs by contamination of mucous membranes/skin abrasions with infected triatomine feces or by ingestion of infective material [2]. Although the contaminative vectorial transmission also occurs, the most common way of infection for mammalian species, as for any other predator in nature, seems to be the oral route through ingestion of infected mammals or triatomines since several carnivore species are avid insect consumers [3]. *Trypanosoma cruzi* exhibits a heterogeneous structure composed of six different discrete typing units (DTUs hereafter) known as TcI-TcVI, which circulate in nature involving all kinds of mammals, including man, and vectors [4]. However, a new entity known as Tcbat has been described. Despite being genetically related to TcI, multiple analyses strongly support the definitive classification of Tcbat as a new DTU [5]. Additionally, this new DTU, initially believed to be strictly associated with bats, has been found to also infect humans [6].

*Trypanosoma cruzi* kinetoplast DNA (kDNA) contains two components (minicircles and maxicircles). The minicircles (1.4 kb long) are abundant (10–20 thousand/cell) and contain different classes present in different ratios in the different *T. cruzi* DTUs [7]. This characteristic of the *T. cruzi* DTUs has been used to genotype *T. cruzi* directly with high sensitivity from biological samples by means of hybridization tests [8–11].

Several species of sylvatic mammals have been found naturally infected with *T. cruzi* in different geographical areas [12–16]. Some of these serve as reservoir hosts of *T. cruzi* as described by Begon [17]. By definition, a parasite species feature in assessing reservoir competence is host infection prevalence, their relative abundance and host infectivity or infectiousness, i.e. infection transmission to a feeding vector [18, 19]. This occurs with the endemic Chilean caviomorph rodent *Octodon degus* [20–22]. This wild rodent has a high relative abundance and has been found highly infected, from 18 up to 70%, only affected by some temporal variations, presumably explained by large-scale global climatic fluctuations [21–23].

By definition, a parasite species that persists in a reservoir host population must have a basic reproduction number (R_0_) equal to or greater than 1. A competent reservoir host is one capable of sustaining a pathogen, a host capable of passing on the infection may not be competent because of the dependence of R_0_ on the abundance of susceptible hosts and on the average life expectancy of infectious hosts (determined by death or recovery) [17, 24].

*Octodon degus* has been found with single or mixed infections with more than two *T. cruzi* DTUs [13, 21, 25]. *Trypanosoma cruzi* DTUs fluctuation was observed in one highly infected *O. degus* followed-up in captivity [26]. The wild vector species in Chile, characterized by mitochondrial sequences, are the endemic *Mepraia* spp., *Mepraia gajardoi* in the north, *Mepraia spinolai* in the central endemic area, and *Mepraia parapatrica* in the intermediate area [27]. The only domestic vector of Chagas disease in Chile is *Triatoma infestans*, which has already been controlled [28]. *Mepraia spinolai* exists in rocky ecotopes associated with small rodents such as *O. degus*. Both, the vector *M. spinolai* and the host *O. degus*, exhibit gregarious behavior, share the same habitats and form large aggregated colonies [22, 29]. Parasitemia in the chronic phase of Chagas disease can be very low, with a detection sensitivity of 1 parasite equivalent/0.2 ml in peripheral blood of a small mammal detected by xenodiagnosis (XD), a sophisticaded method which utilizes the vector acting as a biological culture medium for the detection of *T. cruzi* infection in any kind of host [30]. In the XD non-infected unfed laboratory reared nymphs of triatomines are allowed to feed, during 20–30 min, on the host to be examined. After this time the nymphs become engorged and later, about 30–40 days a pool of feces of the insects is microscopically examined for moving *T. cruzi*. Meanwhile a higher detection sensitivity (0.01 parasite equivalents/ml) is obtained by conventional PCR or qPCR amplifying repeated DNA sequences in blood samples [31, 32]. Previous studies on *T. cruzi* DNA detected in blood samples of *O. degus* via XD, revealed better results when *M. spinolai* is used instead of *T. infestans* to amplify *T. cruzi* minicircle DNA by means of conventional PCR, using triatomine’s intestinal contents as template [33, 34]. The aim of this study is to look for evidence of fluctuations over time in the *T. cruzi* DTUs composition in a group of naturally infected *O. degus*, which may result from natural oscillations in the parasite-vector-reservoir system. The following questions will be addressed using three serial XD followed by PCR assays and *T. cruzi* genotyping: (i) Does *T. cruzi* DTU composition infecting each *O. degus* change over time? (ii) Does *T. cruzi* DTU composition infecting *O. degus* vary over time in XD triatomines? (iii) Does *T. cruzi* DTU composition in *O. degus* change depending on which XD triatomine species is used?

## Methods

### Population under study: *Octodon degus*

Fifty-seven *O. degus* were used for this study (91.6:100 male:female sex ratio), of which only four were juvenile individuals. The animals were captured using traps (H.B. Sherman Trap Company, Tallahasee, FL, USA) in diurnal and nocturnal periods. Each animal was weighed and anesthetized with isofurene at a dose of 13 mg/kg body weight. Once anesthetized, a blood sample of 0.2 ml from the saphenous vein was obtained in the field from each animal to determine infection status. This sample was mixed with a similar volume of solution 6 M Guanidine and 0.2 EDTA pH 8.0 and boiled for 10 min. Animals were ear-tagged and separated into groups of 2–4 rodents of the same sex and kept in acrylic cages. The animals were maintained in an animal room with controlled temperature (25 °C) and relative humidity (45–65%). They received a mixture of three pellets and a complement of fresh vegetables, fruits and water ad libitum. The animals were inspected every 24–48 h. Three animals died during the follow-up study and were necropsied immediately after death.

### Xenodiagnosis

Both *M. spinolai* and *T. infestans* II-III nymphal stages were grown in insect colonies of the Faculty of Medicine, University of Chile. About 4–8 nymphs of each species were fed each time for 30 min and later inspected visually to check they had engorged. A pool of insects of the same species were weighed before and after feeding to record the amount of blood ingested. Three XD, separated by a period of 6 months each were performed. The first XD was performed four months after animals were collected in the field.

### Triatomine feeding with *Mus musculus* and fecal/urine sample collection

*Mepraia spinolai* and *T. infestans* nymphs used for XD were fed using the *O. degus* individuals under study and maintained separately inside a climate chamber at 27 °C with relative humidity of 70% and 14:10 h light:dark photoperiod. Forty, eighty and one hundred and twenty days after feeding with *O. degus*, a pool of feces from all the insects of each vector species was obtained spontaneously after feeding the triatomines using non-infected *Mus musculus* (2% thiopental). Each pool of fecal/urine contents was mixed with 200 μl of double-distilled water, boiled for 10 min, submitted to DNA extraction using a commercial kit (EZNA Blood DNA Mini Kit OMEGA biotek, Nercross, GA) eluted in 100 μl of double-distilled water, and later frozen at −20 °C ready to perform a PCR assay.

### Blood and xenodiagnosis samples analyses

Both blood samples collected from each *O. degus* at the beginning of the study and fecal/urine samples from triatomine of the three XD, each one with analyses after 40, 80 and 120 days of feeding with *M. musculus*, were used to perform the PCR assays.

### PCR assay test

Conventional PCR was performed as previously reported using primers 121 and 122 directed to amplify the variable region of minicircle DNA [35]. Each assay included positive and negative controls. Samples were tested at least three times, and to improve the method’s sensitivity when resulted negative, the DNA extract was concentrated up to four times by evaporation and PCR assayed again. A sample was considered positive when at least one of these assays handed a positive result (presence of amplicon). The qPCR was performed using primers cruzi 1 and cruzi 2 directed to a DNA satellite under the conditions described in [36].

### Genotyping test: *T. cruzi* DTUs

For genotyping, PCR-DNA for blot analyses was performed by using 10 μl of each PCR product. Four *T. cruzi* clones (sp 104 cl 1, CBB cl 3, NR cl 3, and V195 cl 1), corresponding to TcI, TcII, TcV and TcVI, respectively, were used to generate DTU specific probes. Construction of minicircle probes and radiolabeling was performed as described [10]. The PCR products were subjected to electrophoresis, transferred onto Hybond N + nylon membranes (Amersham, Piscataway, NJ, USA), and cross-linked by ultraviolet light for DNA fixation. After transferring PCR products, four membranes were pre-hybridized for at least 4 h at 55 °C. Each membrane was then hybridized overnight with a DTU-specific DNA probe labeled with 32P (1 × 10^6^ cpm/membrane). After hybridization, membranes were washed under high stringency conditions and then exposed using the Molecular Imager FX (Bio-Rad Laboratories, Hercules, CA, USA). DNA amplicons (30–300 ng DNA) were electrophoresed onto an agarose gel 2% after which the DNA was denaturized and later transferred to nylon membranes. Four copies of identical membranes containing DNA blots were hybridized against a panel of the DNA probes specific to recognize specifics lineages of Tcl, TcII, TcV and TcVI, which are the representative *T. cruzi* DTUs circulating in Chile as previously described [9, 13, 37].

### Statistical analyses

We determined whether differences could be detected between the amount of detected DTUs and the weight and sex of the individuals, and the number of parasites detected by means of qPCR. To perform these analyses the program GraphPadPrism 7.0 for Windows was used. One-way ANOVA, Friedman and/or *χ* ^2^ tests were performed depending on the nature of data.

## Results

### Determination of parasitemia by qPCR and *T. cruzi* DTUs composition

**Fig. 1:**
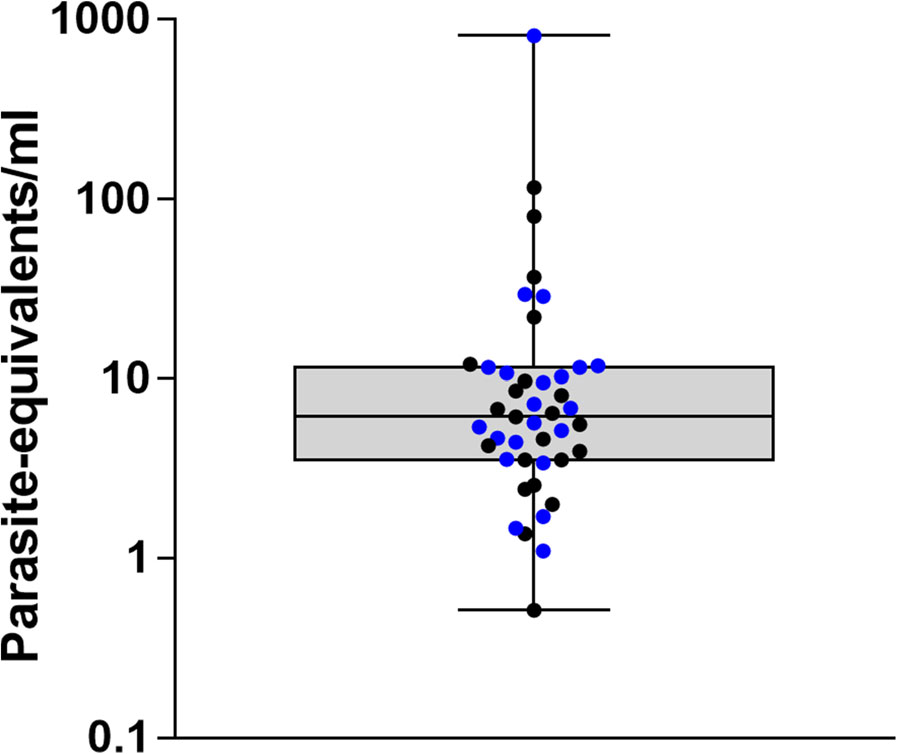
Results of qPCR. Parasite-equivalents/ml of blood for males (*blue dots*) and females (*black dots*)

Immediately after capture, twenty-nine out of the forty infected animals were evaluated for quantification of parasite-equivalents/ml of blood. Figure 1 shows parasite loads of animals immediately after their captures. One sample gave a minimum of 1 parasite equivalent/ml, another gave a maximum of 812 parasite equivalents/ml. The median was 6.2 parasite equivalents/ml and the average was 43 parasite equivalents/ml. The *T. cruzi* DTU composition in nineteen animals revealed single and mixed infections with a preponderance of TcV, and TcVI as the less represented (Table 1).

**Table 1:** DTUs detected by PCR from blood samples, and from XD coupled with PCR (1XD, 2XD and 3XD), per individual *Octodon degus* analyzed

							1XD			2XD			3XD	
Animal	Sex	Weight	qPCR (parasite equivalent/ml)	Inicial blood	Triatomine XD	40 days	80 days	120 days	40 days	80 days	120 days	40 days	80 days	120 days
2	F	112.4	3.54	+	Ms	II-V-VI	II-V	−	I-V	−	−			
					Ti	II-V	II-V	−	+	V-VI	−			
3	M	172	4.4	I-II-V-VI	Ms	II-V	II-V	+	+	−	−	V	I	+
					Ti	II-V	II-V	−	+	−	−	I-V	I	−
7	F	123.7	1.37	VI	Ms	−	−	+	+	+	+	V	V	V
					Ti	−	−	−	−	V	−	V	V	V
8	F	184	3.54	II-V-VI	Ms	II-V-VI	II-V	+						
					Ti	−	II-V	−						
10	M	191	14.728	II-V-VI	Ms	II-V	+	−	+	−	II			
					Ti	II-V	−	−	+	−	−			
13	F	166	0.5114	II-V-VI	Ms	II-V	+	−	−	−	+	−	V-VI	V
					Ti	II-V	−	−	−	V	−	V	−	V
22	F	132	21.94	II-V-VI	Ms	−	−	−	+	−	V	+	V-VI	−
					Ti	−	−	−	+	−	−	VI	−	−
25	F	134	4.24	+	Ms	−	−	−	+	−	−	−	−	−
					Ti	+	−	−	+	−	−	−	−	−
29	M	187	10.26	II-V	Ms	II-V	+	−	−	−	+	−	−	−
					Ti	II-V	−	−	+	−	+	−	−	V-VI
31	F	149	3.96	V-VI	Ms	−	−	−	+	−	−	−	−	−
					Ti	−	−	−	−	−	−	−	−	−
32	F	201	5.56	V	Ms	−	−	−	+	−	−	−	−	−
					Ti	−	−	−	+	−	−	−	−	−
33	F	196	2.56	V-VI	Ms	+	−	−	+	−	−	−	−	−
					Ti	−	−	−	+	−	−	−	−	−
34	F	184	6.78	II	Ms	−	+	+	+	−	V	−	−	+
					Ti	−	+	+	+	V	V	−	−	I-V
40	M	133	11.54	V	Ms	+	+	+	−	−	+	−	+	I-II-V
					Ti	+	+	+	+	−	+	−	I	I-II-V
41	M	120	5.68	II-V	Ms	+	+	−	−	−	+	−	−	−
					Ti	+	+	−	+	V	−	−	−	−
42	M	171	9.5	II-V	Ms	−	−	+	−	−	I	−	−	−
					Ti	+	+	+	−	+	+	−	−	−
43	M	107	10.72	II-V	Ms	+	+	V	+	V	+	+	I-II-V	I-V
					Ti	−	+	−	+	+	+	II	I-II	−
50	M	125	812.2	I-II	Ms	+	+	V	V	+	I	−	II	−
					Ti	+	+	−	−	+	+	−	I-II-V	−
51	M	129	7.24	II-V	Ms	I-V	I	−	V	+	−	−	V	−
					Ti	I-V	V	−	+	+	−	V	−	−
52	M	141	28.74	II-V-VI	Ms	I-V	−	−	V	+	−	−	+	−
					Ti	I-V	I-V	−	+	+	−	−	+	−
53	F	122	116.2	+	Ms	I-V	−	−	−	+	−	V	−	−
					Ti	I-V	−	−	+	+	+	V	−	−
54	F	163	80.28	+	Ms	−	−	−	+	−	−	V	−	−
					Ti	−	−	−	−	−	+	V	−	−
57	M	117	5.14	II-V	Ms	−	−	−	+	+	−	V	−	+
					Ti	I-V	−	−	+	−	+	−	+	+

*Abbreviations: F* female, *M* male, *Ms* *Mepraia spinolai*, *Ti* *Triatoma infestans*

The “–” sign indicates a negative conventional PCR result. The “+” sign indicates a positive PCR result, but these samples did not hybridize with any of the probes used. The dark lockers indicate that the individual perished before performing the XD. Analyses 1, 2 and 3 correspond to post-XD feeding with *Mus musculus* at 40, 80 and 120 days, respectively

### Serial xenodiagnosis on *Octodon degus*

*Octodon degus* (23 in total) were followed-up after the three XD to gather information on the infective *T. cruzi* DTUs composition. In the case of other four *O. degus*, even though the infective status was demonstrated, no information about *T. cruzi* DTUs was obtained since the PCR amplicons had insufficient DNA (under 30 ng) to genotype or the infective genotype belonged to an unknown *T. cruzi* DTU (Table 1). Three animals died during the study (one after the first XD and two after the second XD). The overall information after the follow-ups on the animals regarding *T. cruzi* DTU composition rates TcV as the most prevalent (56%, with 70 total detections), followed by TcII (20%, with 25 total detections) and TcI (18.4%, with 23 total detections), and finally TcVI (5.6%, with 7 total detections) in the circulating blood and amplified in the guts of the XD triatomines (Table 1). The most frequent infections after the whole follow-up are mixed infections of DTUs I, II and VI (one case), DTUs I, II and V (four cases), DTUs II, V and VI (three cases), DTUs I and V (four cases), DTUs II and V, and DTUs V and VI (one case each). The only single infections were TcV in three cases and, TcI in one case. Fluctuations on *T. cruzi* DTUs are observed in several *O. degus* followed in more than one XD (changes in DTU composition) as observed in animals number 2, 3, 13, 22, 29, 34, 43, 50, 51, 52, 53 and 57. These animals represent over 50% of the total infective 23 infective animals studied.

The fluctuations of DTU composition were mainly of mixed infections with two DTUs to single infections, or vice versa. However, in few cases fluctuations of mixed infections with three DTUs were observed in the first and third XD (animals numbers 13, 29, 43 and 50), and fluctuations with four DTUs were observed in the first and second XD (animals numbers 2 and 3). Despite obtaining confirmation of their infective status, it was not possible to determine *T. cruzi* DTU composition for four animals (animals numbers 25, 31, 32 and 33). Three animals died during the follow-up as indicated in Table 1. It was not possible to determine the cause of death of animals numbers 2 and 10. Meantime the animal number 8 presented a dilated heart and mega colon with coprolites. The serial XD in several *O. degus* evidenced fluctuations in parasitemia over time. There were cases in which at one time due to a high level of parasitemia both triatomine species became infected with more than one *T. cruzi* DTU, and later no infection or no *T. cruzi* DTUs was detected, or vice versa. *Octodon degus* numbers 22, 29, 34, 40, 52, 53 and 57 represented typical cases with maximal parasitemia fluctuations. However, there were cases with high parasitemia over time with fluctuations in the *T. cruzi* DTU composition (animals numbers 2, 3, 50). Other cases presented *O. degus* with very low parasitemia over time (animals numbers 25, 31, 33, 41, 42 and 54). In these cases, it was possible to detect the presence of *T. cruzi* DNA but not to determine the infective *T. cruzi* DTU composition due to insufficient DNA to perform genotyping or the presence of unknown DTU. The determination of *O. degus* parasitemia before serial XD by qPCR revealed a great heterogeneity, i.e. between 1 and 812 parasite equivalents/ml of blood. The results on qPCR and serial XD indicate that parasitemia on the studied animals fluctuated over time between qPCRs and XDs. Quantification by qPCR determines the amount of parasite equivalents/ml making the detection of high and low levels of parasitemia possible, high levels of parasitemia allow for the determination of DTU composition while low levels only serve to determine the infective status of the animal. Additional parasitemia in the animals was estimated by the serial XD followed by conventional PCR on the infected triatomine samples. These estimates are the minimal figures based on the total amount of blood ingested from both triatomine species pool and the number of different *T. cruzi* DTUs detected by them. Both insect species ingest an average of 150 μl of blood. The minimal theoretical parasitemia is 7 parasite equivalents/ml when one single parasite infects only one insect. When both insect species became infected that minimal parasitemia would fringe to 14 parasite equivalents/ml. However, when both insect species became infected with two different DTUs that minimal parasitemia would be over 28 parasite equivalents/ml and much higher when three DTUs are detected by both species.

### Serial evaluations of infectivity and *T. cruzi* DTU detection over time with triatomine xenodiagnosis

**Fig. 2:**
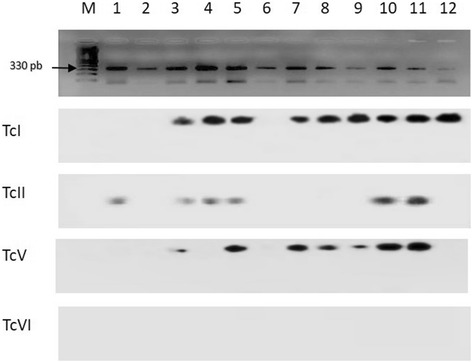
*Trypanosoma cruzi* DTUs present in *Triatoma infestans* (Ti) and *Mepraia spinolai* (Ms)*.* Southern blot with kDNA PCR of the intestinal dejection of both vectors after successive events of laboratory feeding. Four representative probes of *T. cruzi* DTUs were used. The results are indicated in the following rows as TcI, TcII, TcV and TcVI. Lane M: DNA ladder; Lane 1: Animal # 43, 3XD after 40 days, Ti; Lane 2: Animal # 40, 3XD after 80 days, Ms; Lane 3: Animal # 43, 3XD after 80 days, Ms; Lane 4: Animal # 43, 3XD after 80 days, Ti; Lane 5: Animal # 50, 3XD after 80 days, Ti; Lane 6: Animal # 34, 3XD after 120 days, Ms; Lane 7: Animal # 34, 3XD after 120 days, Ti; Lane 8: Animal # 2, 2XD after 40 days, Ms; Lane 9: Animal # 3, 3XD after 40 days, Ti; Lane 10: Animal # 40, 3XD after 120 days, Ms; Lane 11: Animal # 40, 3XD after 120 days, Ti; Lane 12: Animal # 40, 2XD after 80 days, Ti. *Abbreviations*: Ti, *Triatoma infestans*; Ms, *Mepraia spinolai*

Both triatomine species exhibited almost the same results for infectivity, *M. spinolai* with 90 and *T. infestans* with 85 positive cases and/or DTU composition (*M. spinolai* with 10 TcI, 12 TcII, 34 TcV and 4 TcVI *vs* *T. infestans* with 12 TcI, 12 TcII, 34 TcV and 2 TcVI) even though the average of blood ingested by *T. infestans* nymphs was greater than the blood ingested by *M. spinolai* nymphs, 93 μl *vs *55 μl, respectively (Table 1). Most of the gathered information on infectivity and *T. cruzi* DTUs composition was obtained in the first (40 days) rather than at the second and third time (80–120 days) post-infection or triatomine feeding on *O. degus* (infectivity detection: 75, 56 and 44 cases over the first, second and third insect evaluations, respectively) (*χ* ^2^ = 16.118, *df* = 2, *P* = 0.0003). The amount of cases in which DTU detection was possible was of: 56, 57 and 44 cases over the first, second and third insect evaluations, respectively. Some results of genotyping were obtained at the third analysis (120 days post-infection) and not in the previous ones, this being the case for XD_1_ *O. degus* numbers 43 and 50; XD2 *O. degus* numbers 22, 34 and 42 and XD3 *O. degus* numbers 29, 34 and 40. In these cases, the parasites required more time to flourish in the midgut. One special and singular case was observed with animal number 50 and *M. spinolai* XD, which harboured different DTUs at the first and the third evaluation. Figure 2 shows representative results on infectivity (presence of amplicon), and *T. cruzi* DTUs composition (hybridization tests with the four *T. cruzi* DTU specific probes). This figure also shows that the amplicon signal can exhibit differential intensities.

### *Trypanosoma cruzi* detection at capture and after serial xenodiagnosis

The number of *T. cruzi* DTUs present in the initial blood sample and after the third XD-PCR (Table 1) were analyzed using the Friedman test. We found a decrease in the number of detected *T. cruzi* DTUs when comparing blood samples and XD samples (Friedman statistic: 16.19, *P* = 0.0010). However, we did not find that decrease for *T. cruzi* DTUs between the different XD (Friedman statistic: 2.413, *P* = 0.2993).

### Statistical analyses on qPCR, body size, sex and DTUs

We did not find statistically significant differences between the number of detected DTUs and the number of parasites detected by means of qPCR (*P* = 0.8868). In addition, we did not find statistically significant differences between the number of detected DTUs and the weight of *O. degus* individuals (*P* = 0.5170). The low number of juveniles (*n* = 4) did not allow us to perform comparisons with adults of *O. degus*. We did not find statistical differences between sexes in the detected DTUs (*P* = 0.0623).

## Discussion

Parasitemia in mammals can be very low in the case of chronic Chagas disease patients, where fluctuations of circulating *T. cruzi* are expected. Therefore, there is a need to perform serial determinations to obtain more sensitive parasitological diagnosis [38, 39]. In a previous report, temporal fluctuations of parasitemia with different *T. cruzi* DTUs was observed in one out of two naturally infected *O. degus* studied by means of serial XD-PCR [26]. The longitudinal study performed in this study with 23 naturally infected *O. degus* submitted to three serial XD, using two vector species, analyzed three times by means of serial PCR assays confirms and extends the previous study now with a larger sample size. We have modified the classical XD of microscopic observation and replaced it with XD-PCR that includes permanent triatomine artificial feedings after infection to provide enough nutrients and allow cases with minimal trypanosome burden to proliferate. We found temporal fluctuations of *T. cruzi* DTUs composition in the animals, with DTU detection (higher parasitemia) to cases of infections without DTU detection (lower parasitemia) or vice versa. Other cases involved fluctuations of DTU composition within the same animal. The gathered information collected from all the XD is that *O. degus* were infected with four, three and two different DTUs at the same time. Only few animals resulted infected with only one *T. cruzi* DTU. However, the circulating parasite composition was less complex when XD-PCR was performed only once or when the triatomines were analyzed at a single time. Most of the results were obtained with insect analysis performed 40 or more days post-infection. However, there are XD triatomines from which information about *T. cruzi* infections and/or DTU composition was obtained 120 days post-infection suggesting cases of low parasitemia at the time XD was performed. These results indicate that *O. degus* has different host infectivity at different times, and the reasons deserve to be studied.

In this study, a prevalence of 75% of *T. cruzi* infection in *O. degus* was found. A previous study of *T. cruzi* prevalence in *O. degus* during four consecutive years in the same endemic area of this study by means of *T. cruzi* detection using one single determination in peripheral blood of *O. degus*, varied over the years (between 18 and 70%) [21]. The resulting *T. cruzi* DTU composition consisted of equivalent rates of single and mixed infections; however, the relative importance of each DTU changed among years, TcV being the most represented. Most of mixed infections consisted of a combination of two *T. cruzi* DTUs [21]. The results of the present follow-up study show a higher rate of mixed infections, which were composed of two, three and four DTUs (15 animals), with TcV as the most represented. Single infections were detected in only four animals. These results demonstrate that a longitudinal study increases the sensitivity of *T. cruzi* DTU detection in animals. Even though in the present study with serial determinations we detected mostly mixed infections with three of the four *T. cruzi* DTUs studied, probably other unknown DTUs are also infecting these animals. It is worth noting that the temporal fluctuations of the observed *T. cruzi* DTUs can be explained either by elimination of one specific *T. cruzi* DTU, or by the possibility of a *T. cruzi* DTU not being accounted due low parasitemia and the method’s sensitivity limit which makes parasite detection impossible. The temporal fluctuations of the four *T. cruzi* DTUs reported here could be explained by permanent colonization of different tissues and release of *T. cruzi* into the vascular system. However, it is also expected that parasitemia could be permanently controlled by the immune system. This equilibrium can be changed in immunocompetent hosts submitted to several kinds of stress. This could be the case of the rodents used in this study, which were submitted to a stress condition after being captured from the wild environment, transported to the laboratory, and later submitted to an acclimatization process under controlled laboratory conditions. Evidence to the general assumption that when an animal is under stress, its resistance to infection decreases, since inflammatory responses would diminish due to increase in glucocorticoid levels [40, 41]. For example, animals infected with *Trichomonas* sp. showed an increase in the number of parasites for about two weeks after being submitted to stress factors. After this period the number of parasites decreased possibly due to the stress adjustment [41]. We suggest that the tendency for a decrease in the number of *T. cruzi* DTUs in the observed mixed infections comparing initial blood samples with XD samples can be explained by the host immune system. Alternatively, the vectors can filter some *T. cruzi* DTUs, or a combination of both. Results of this kind have been obtained in infected dogs stressed by altering their nutritional state and studied by means of XD. Dogs with bad nutritional state presented much higher levels of infecting triatomine XD [42]. Our results confirm that *O. degus* is a competent reservoir [43]. This rodent presents a high prevalence of infection with *T. cruzi*, lives in colonies located close to the vector *M. spinolai* [20, 22] and has high infectiousness to triatomines [44]. In this study, we support the idea that the two analyzed vector species are equally competent to maintain *T. cruzi*.

Our results confirm that circulating *T. cruzi* DTUs in *O. degus* can be quite different in a longitudinal study [26]. In another study using the same two triatomine species used here, *M. spinolai* showed a better susceptibility than *T. infestans* to amplify *T. cruzi* from naturally infected *O. degus* [33]. The reason for this inconsistency remains unknown but *T. infestans* specimens used in [33] and the present study pertained to the same insect colony established since 1950. Meantime the *M. spinolai* colonies used varied between the two studies; probably some of them have a higher susceptibility to become infected. Regarding the parasitemia level of naturally infected *O. degus*, we estimate that such figure should be much higher than 1 parasite/0.15 ml, which is the minimal theoretical amount to be detected in the 150 μl of blood intake (7 parasite equivalents/ml). We estimate that parasitemia in animals with one single *T. cruzi* DTU is probably 5–10 times higher than the minimal theoretical amount for each XD that is 35–70 parasite equivalents/ml.

Higher parasitemia can be expected when both insect species are infected with mixed *T. cruzi* DTUs. Parasitemia of the *O. degus* studied by qPCR varied from less than 1 to 812 parasite-equivalents/ml with a median of 6.16 parasite-equivalents/ml of blood. Parasitemia in human chronic cases has been estimated in different countries by means of qPCR [45, 46]. The obtained medians fluctuated between 1.27–2.38 parasite equivalents/ml. In the meantime, the established medians for dogs and cats were 8.1 and 9.7 parasite equivalents/ml, respectively [47]. To overcome these limitations of parasite detection sensitivity, and to study the dynamics of circulating *T. cruzi* DTUs in a small rodent such as *O. degus* it is necessary to amplify first the infecting *T. cruzi* by means of XD, to further amplify *T. cruzi* DNA with PCR assays and later characterize them. In this way, using XD we are also confident that living parasites have been studied and results are not due to lysed ones. This result resembles the finding of infectiousness of *T. infestans* by infected dogs and cats which increased steeply with parasitemia. Dogs and cats with 80% of infectiousness on *T. infestans* displayed median parasite load determined by qPCR of 21.3 or 96.1 parasite equivalents/ml, respectively [48]. In summary, using a combination of XD and PCR assays, this study reports for the first time the occurrence of temporal fluctuations of *T. cruzi* DTU composition in a large sample of naturally infected *O. degus* reservoirs. We showed that it is possible to analyze complex mixtures of circulating *T. cruzi* DTUs using two vector species in the XD over time. The time necessary to detect easily infected triatomines ranges from 40 to 120 days. We also conclude that *M. spinolai* and *T. infestans* are equally competent to maintain several *T. cruzi* DTUs.

## Conclusion

Serial XD coupled with *T. cruzi* DTU identification (TcI, TcII, TcV, TcVI) by means of conventional PCR and hybridization tests, provided evidence for different DTUs composition of *T. cruzi* circulating in peripheral blood over time. The XD performed with the vectors *M. spinolai* and *T. infestans* reveals that *O. degus* infects both insect species, confirming that *M. spinolai* and *T. infestans* are equally competent to maintain *T. cruzi* DTUs, since similar results of infection were obtained after xenodiagnosis procedure. XD triatomines analyzed 40 days post - infection resulted the optimum to evaluate infectiveness rather than insects analyzed at 80 or 120 days. *Trypanosoma cruzi* parasitemia is estimated based on XD triatomines able to detect infection and/or *T. cruzi* DTUs composition. In addition, an evaluation of parasitemia was performed before the serial XD study by means of qPCR.

## Abbreviations

DNA: Deoxyribonucleic acid
DTUs: Discrete typing units
kDNA: Kinetoplastidic DNA
PCR: Polymerase chain reaction
PCR-XD: Conventional PCR performed with fecal samples of XD
qPCR: quantitative PCR
XD: Xenodiagnosis
